# Phosphorus‐Based Anodes for Fast Charging Lithium‐Ion Batteries: Challenges and Opportunities

**DOI:** 10.1002/smsc.202200015

**Published:** 2022-04-21

**Authors:** Hongchang Jin, Yingshan Huang, Chaonan Wang, Hengxing Ji

**Affiliations:** ^1^ Hefei National Laboratory for Physical Sciences at the Microscale CAS Key Laboratory of Materials for Energy Conversion School of Chemistry and Materials Science University of Science and Technology of China Hefei 230026 China

**Keywords:** fast charging, lithium-ion batteries, lithium-ion diffusion, phase transitions, phosphorus

## Abstract

Building better lithium‐ion batteries with higher power density is critical to enhancing the operational experience of portable electronics and electric vehicles. The factors that limit power density at the cell level are a lower rate capability of the anode than the cathode and lithium plating at the anode when recharging at a high rate that increases the risk of internal short circuit and creates a safety hazard. Therefore, developing new anode materials with high rate performance with low lithium plating risk is the key to improve the power density and at the same time achieving extremely fast charging capability. Herein, a comparative review on the advantages and challenges in using graphite, silicon/graphite, and the newly emerging phosphorus‐based anodes, for fast charging, is presented.

## Graphical abstract

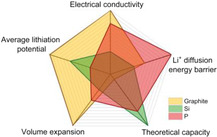

## 1 Introduction

Because of their high energy density and cycling stability, lithium‐ion batteries (LIBs) are indispensable components of portable electronics and electric vehicles (EVs).^[^ ^1^, ^2^ ^]^ Present‐day commercial LIBs have energy density of ≈280 Wh kg^−1^ at the cell level, which allows the driving range of EVs to exceed 600 km. However, LIBs typically require about 1 h for a full recharge; for example, a Porsche Taycan takes ≈20 min to charge from 20% to 80% state of charge (SOC), and a Tesla Model 3 needs ≈30 min to charge from 0% to 80% SOC.^[^ ^3^, ^4^ ^]^ These charging times are much longer than the time required to refuel internal combustion engine‐driven vehicles (3–5 min). The long recharging time of current LIBs is the principal limitation when using LIBs in portable electronics and EVs. Thus, it is highly desirable to develop LIBs having “extremely fast charging” capability (XFC, which is defined as the ability of recharge up to 80% of the battery capacity in 10 min or less)^[^ ^5^ ^]^ but at the same time being safe and having a long lifespan. In XFC, LIBs are subjected to a charging rate of 6 C, which requires both the cathode and anode to have a high rate capability for fast lithium storage. Recent studies on the rate capabilities of graphite|LiNi_0.8_Mn_0.1_Co_0.1_O_2_ pouch cells have revealed that in these cells, the anode shows only 28% capacity retention at 6 C, which is considerably lower than that of the cathode (78%), indicating that the anode has a much lower rate capability than the cathode.^[^ ^6^ ^]^ Moreover, fast charge is accompanied by increased electrode polarization, which leads to a much lower working potential of the anode than when charging at moderate rates. Thus, under XFC, the anode potential may be lowered to <0 V versus Li/Li^+^, resulting in Li metal plating at the anode surface, which creates a safety hazard in the form of an internal short circuit. Thus, at the cell level, the limited rate capability and risks associated with Li plating of the anode are the two important factors that limit XFC of LIBs.

**Figure 1:**
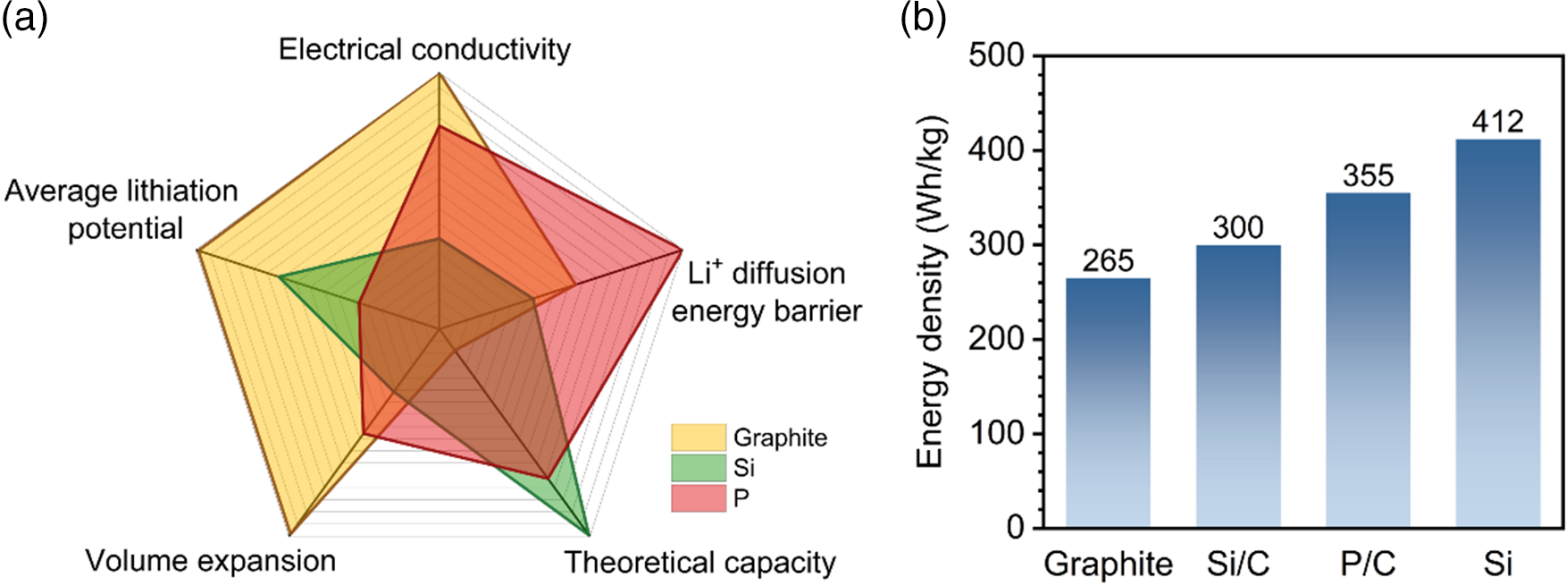
A comparison of graphite, Si, and phosphorus anode materials: a) gravimetric energy density, average lithiation potential, volume expansion, theoretical capacity, Li‐ion diffusion barrier, and electrical conductivity (black phosphorus was used as the P‐based anode material in this panel); b) gravimetric energy density of graphite, Si/graphite (Si/C, Si content <20 wt%), phosphorus/carbon‐based anode (P/C, P content >50 wt%), and Si in full cell. We applied these anode materials together with nickel–cobalt–aluminum (NCA) cathode in 2170 cells (basic power units in 2019 Tesla Model 3 Performance car). We consider a constant electrode volume of 13.00 cm^3^ in calculation since the standard 2170 cell has a known space to contain electrodes, current collector, separator, and electrolyte. The densities of Si and P/C anodes were taken to be 1.5 g cm^−3^, while that of both graphite and Si/C anodes was 1.6 g cm^−3^.

Graphite has been the uncontested material of choice in most commercial LIBs since their commercialization 25 years ago. Graphite is favored due to its high capacity (372 mAh g^−1^), low lithiation potential (≈0.1 V vs. Li/Li^+^), and excellent reversibility during repeated Li intercalation/deintercalation (**Figure** **1**a).^[^ ^7^ ^]^ With the growing demand for high‐energy‐density LIBs, graphite is being gradually replaced by Si/graphite composites (Si content <20 wt%).^[^ ^8^ ^]^ Since silicon has a high theoretical capacity (4200 mAh g^−1^) and low lithiation potential (≈0.2 V vs. Li/Li^+^), the Si/graphite composite anode can yield a cell‐level energy density of >300 Wh kg^−1^ when coupled with an appropriate cathode material (Figure 1b).^[^ ^9^ ^]^ Nowadays, the energy densities of LIBs are high enough to meet the requirements of most application scenarios and this makes their XFC capability more important.

Here, we revisit the above‐described anode materials with regard to XFC capability. Ideally, anodes for XFC should have favorable Li‐ion diffusion capability coupled with good electron conductivity. Commercial graphite has high values for both electrical conductivity (10^6^ S m^−1^) and an Li‐ion diffusion coefficient (10^−11^–10^−7^ cm^2^ s^−1^),^[^ ^10^ ^]^ but electrolysis of the electrolyte at the graphite surface forms a solid–electrolyte interphase (SEI), which impedes Li‐ion diffusion into the graphite interlayer. Thus, the nature of the interphase is the major limiting factor of graphite anodes for fast lithium storage. Silicon has a relatively low electrical conductivity (≈10^−3^ S m^−1^) and low Li‐ion diffusion coefficient (10^−16^ cm^2^ s^−1^),^[^ ^11^, ^12^ ^]^ both of which are not favorable for fast charging. Silicon also undergoes ≈400% volume expansion during lithiation, which causes massive SEI growth and results in the active material losing electrical contact, which leads to capacity loss and impedes electron/ion transport. In addition, both graphite and Si/graphite work at lithiation/delithiation potentials <0.2 V versus Li/Li^+^. Even though a low working potential of anode is required for LIBs to output an average voltage of >3 V when coupled with oxide cathodes, fast charging induces electrode polarization that effectively lowers the anode potential to below that required for Li plating. Therefore, the development of new anode materials systems with both high energy density and XFC capability is of great interest. Transition metal compounds (such as M_*x*_S_*y*_, M_*x*_P_*y*_, Fe_*x*_O_*y*_, M = Fe, Mo, Co, etc.) have been proposed to be an excellent potential replacement for graphite due to high theoretical capacity (2–3 times of graphite) and moderate electronic conductivity (higher than Si).^[^ ^13^, ^14^, ^15^, ^16^, ^17^ ^]^ However, the high average delithiation potential (higher than 1 V vs. Li/Li^+^) of transition metal compounds reduces the gravimetric energy density in the full cell. Most recently, phosphorus‐based anode materials have drawn much attention for their capability for fast charging.^[^ ^18^, ^19^ ^]^ Phosphorus (we use black phosphorus as an example here) has a good electrical conductivity of 300 S m^−1^, a low Li diffusion barrier of 0.08 eV (lower than graphite, 0.33 eV, and Si, 0.58 eV),^[^ ^20^, ^21^, ^22^ ^]^ and a high gravimetric capacity (2596 mAh g^−1^, Figure 1b), which helps offset its relatively high voltage loss versus (Li/Li^+^) (≈0.7 V on average) to render a high gravimetric energy density, all of which make phosphorus a candidate material for an LIB anode with respect to XFC targets. However, similar to most anode materials having a high theoretical capacity, for example, silicon, the Li alloying–dealloying reaction of phosphorus is accompanied by complex phase changes, a large volume expansion, as well as a low initial coulombic efficiency and cycling stability.

In this perspective, we discuss the structure and interphase properties of graphite, Si/graphite, and phosphorus‐based anode materials for LIBs. Based on our observations and those reported in the literature, we discuss the key challenges and describe possible strategies to obtain efficient anode materials for XFC LIBs. Finally, we highlight the unique advantages of phosphorus‐based anodes in achieving XFC as compared with graphite and Si/graphite.

## 2 Graphite Anode

**Figure 2:**
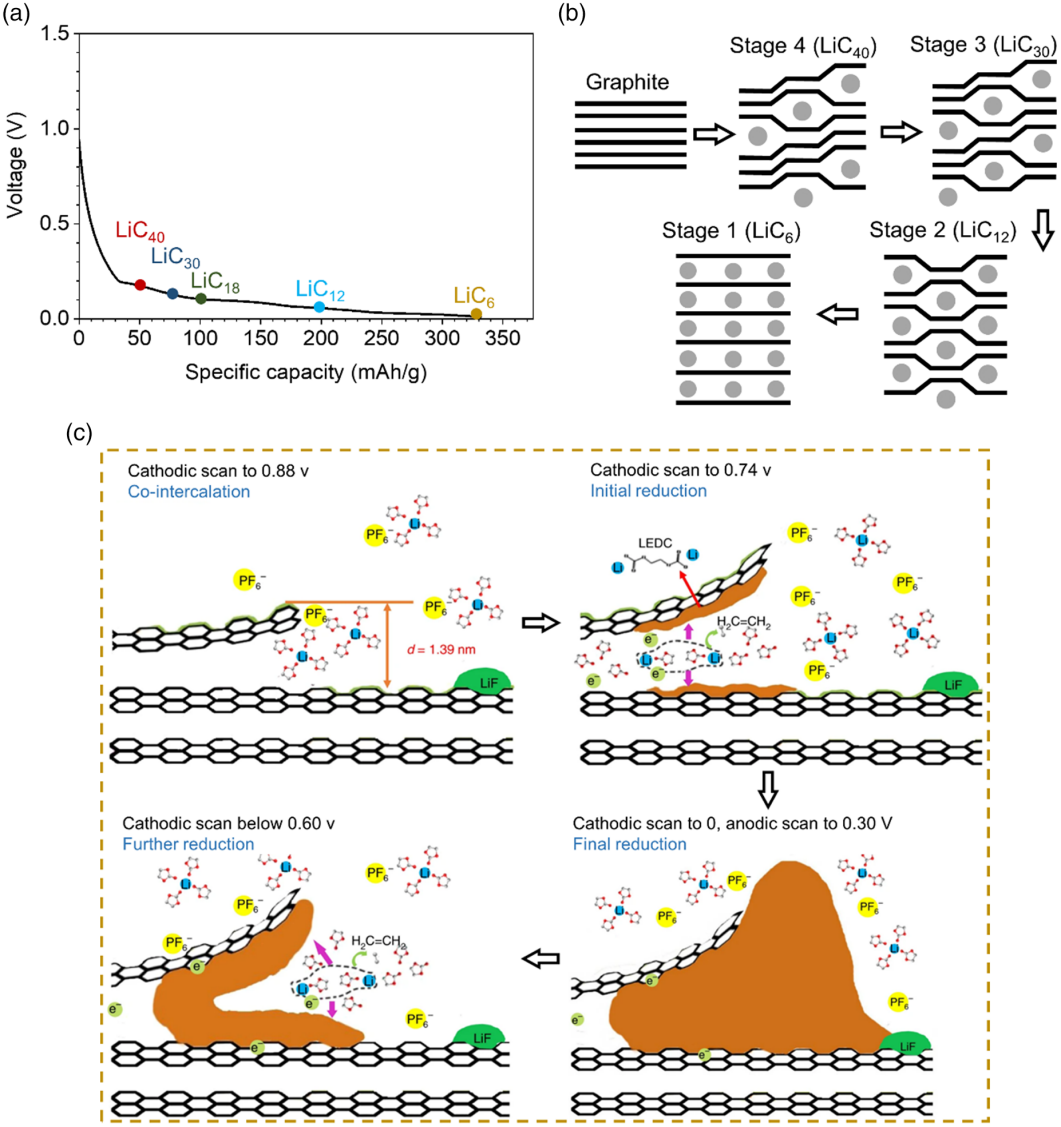
a) Lithiation curves of graphite. b) Phase changes of graphite during Li intercalation. c) Schematic illustration of the interphase formation chemistry during the very first lithiation. c) Reproduced with permission.^[^ ^34^ ^]^ Copyright 2019, Springer Nature.

Li storage in graphite is based on intercalation reactions, in which Li ions are intercalated in the interplanar spaces of graphene planes.^[^ ^23^, ^24^, ^25^, ^26^, ^27^ ^]^ The lithiation of graphite occurs through the edge planes of graphite platelets in stages with stages *n* = 1L, 4 (LiC_40_), 3 (LiC_30_), 2 (LiC_12_), and 1 (LiC_6_) (**Figure** **2**a,b).^[^ ^28^, ^29^ ^]^ The complex phase evolution determines the differences in diffusion kinetics during the different stages. The Li‐ion diffusion coefficient (*D*_Li+_) decreases from 10^−6^ to 10^−9^ cm^2^ s^−1^ when the LiC_*x*_ phase transits from LiC_12_ to LiC_6_.^[^ ^30^ ^]^ indicating that high energy barriers need to be overcome in order for this phase transition to occur. For example, the Li migration barrier increases from 308 meV (LiC_30_) to 400 meV (LiC_6_).^[^ ^30^ ^]^ Although there is a high barrier to the lithiation of the graphite anode, the *D*_Li+_ value is still comparable with those of the cathode materials used. The cathode electrodes show lower *D*_Li+_, for example, in the range from 10^−12^ to 10^−13^ cm^2^ s^−1^ for lithium cobalt oxide,^[^ ^31^ ^]^ 10^−14^ to 10^−16^ cm^2^ s^−1^ for lithium iron phosphate,^[^ ^32^ ^]^ and 10^−10^ to 10^−11^ cm^2^ s^−1^ for lithium nickel cobalt manganese oxide.^[^ ^33^ ^]^ These results show that even though graphite has a good Li‐ion diffusion capability across the interlayer, it still shows lower capacity retention than the cathode during XFC in a full cell. Therefore, Li‐ion diffusion across the graphite layers is not likely to be the major factor affecting the fast charging performance of graphite.

In practice, before intercalating in the graphite interlayer, Li ions have to cross the SEI LIBs operation. The electrolyte, for example, ethylene carbonate (EC) and lithium hexafluorophosphate (LiPF_6_), decomposes during the lithiation process, and the decomposition product (SEI) forms a solid coating made up of organic and inorganic products on the surface of graphite. First, PF_6_^−^ decomposes to LiF at 1.5 V (vs. Li/Li^+^), after which, Li‐EC cointercalates into the graphite layer at 0.88 V (Figure 2c), which causes exfoliation of the graphite layer.^[^ ^34^ ^]^ As the potential drops from 0.74 to 0 V, the decomposition of EC gives rise to ROLi and ROCO_2_Li (where R is an alkyl group), which further partially decompose to Li_2_O and Li_2_CO_3_.^[^ ^35^ ^]^ The organic and inorganic products in the SEI have different Li‐ion conductivities; previous studies have indicated that Li‐ion diffusion is faster in the organic layer than in the inorganic layer.^[^ ^36^, ^37^, ^38^, ^39^, ^40^, ^41^, ^42^ ^]^ As the graphite interlayer is the only channel through which Li ions can intercalate into the interlayer spaces in graphite, the complex structure of the SEI greatly influences Li‐ion diffusion from the electrolyte into the graphite interlayer.

The thickness of the SEI increases upon aging, and the repeated Li‐ion/Li‐EC intercalation and deintercalation cause exfoliation of the graphite layer.^[^ ^35^ ^]^ The unstable interphase further reduces Li‐ion diffusion under XFC, which causes the lithiation potential to drop from 0.1 V (vs. Li/Li^+^) to slightly below 0 V, resulting in lithium plating.^[^ ^43^ ^]^ The deposited lithium is not fully removed during the following discharge process and can lead to capacity loss and also raise safety issues. Therefore, at the cell level, interphase optimization is critical to achieving a breakthrough change in the fast charging capability of graphite and also improve safety of the battery. Recent research has found that the addition of Li_3_N in the SEI can effectively improve ion diffusion ability and inhibit the decomposition of electrolyte. Using this strategy, a capacity retention of graphite up to 82.4% can be obtained at 2.5C.^[^ ^44^ ^]^ Moreover, introducing inorganic components that have high thermal stability, such as LiF, Li_2_S, and Li_2_S_2_O_4_, can restrict solvent cointercalation and achieve a high capacity retention of 220 mAh g^−1^ at 4C.^[^ ^45^ ^]^ Amorphous Al_2_O_3_ coating on the graphite surface is an efficient way to increase electrolyte wettability on the graphite and improve the fast charging capability, exhibiting a reversible capacity of about 337 mAh g^−1^ at a high rate of 4 A g^−1^.^[^ ^46^ ^]^ Surface engineering of graphite with a cooperative biphasic MoO_*x*_–MoP_*x*_ promoter exhibits a fast charging capability (<10 min charging for 80% of the capacity) by mitigating the formation of resistive films and lowering the Li^+^ adsorption energy.^[^ ^47^ ^]^ Moreover, purely interfacial modification using solid electrolytes to form an artificial SEI on graphite also works, like Li_3_BO_3_–Li_2_CO_3_, which decreases interphase impedance by >75% and enables 80% capacity after 500 cycles in pouch cells with >3 mAh cm^−2^ area capacity at 4C.^[^ ^48^ ^]^ These results have further demonstrated the importance of the interphase in achieving fast lithium storage in graphite.

Thus, graphite has the merit of having good electrical conductivity and Li‐ion diffusion capability across the interlayer. However, the SEI at the edge plane impedes the transport of Li ions to the diffusion channel of graphite, indicating that the structure of the interphase plays an important role in limiting the rate performance of graphite. In view of these limitations of the currently used graphite in achieving XFC, it has become necessary to search for new alternatives to graphite; in the following, we describe two alternative materials.

## 3 Si (SiO_*x*_)/Graphite Anode

Due to its high theoretical capacity, the silicon anode has received much attention in recent years. Each silicon atom can store 4.4 lithium atoms resulting in a theoretical capacity of 4200 mAh g^−1^; however, this process is accompanied by a very large volume expansion. Usually, silicon is mixed with graphite to achieve a balance between capacity and stability and also to compensate for the poor electrical conductivity of Si. A high silicon content can cause the overpotential to increase and therefore, quasicommercial Si/graphite anodes usually contain less than 20 wt% silicon.^[^ ^49^ ^]^ To evaluate the fast charging performance of Si/graphite anode, we first focus on the Li‐ion diffusion capability of silicon.

**Figure 3:**
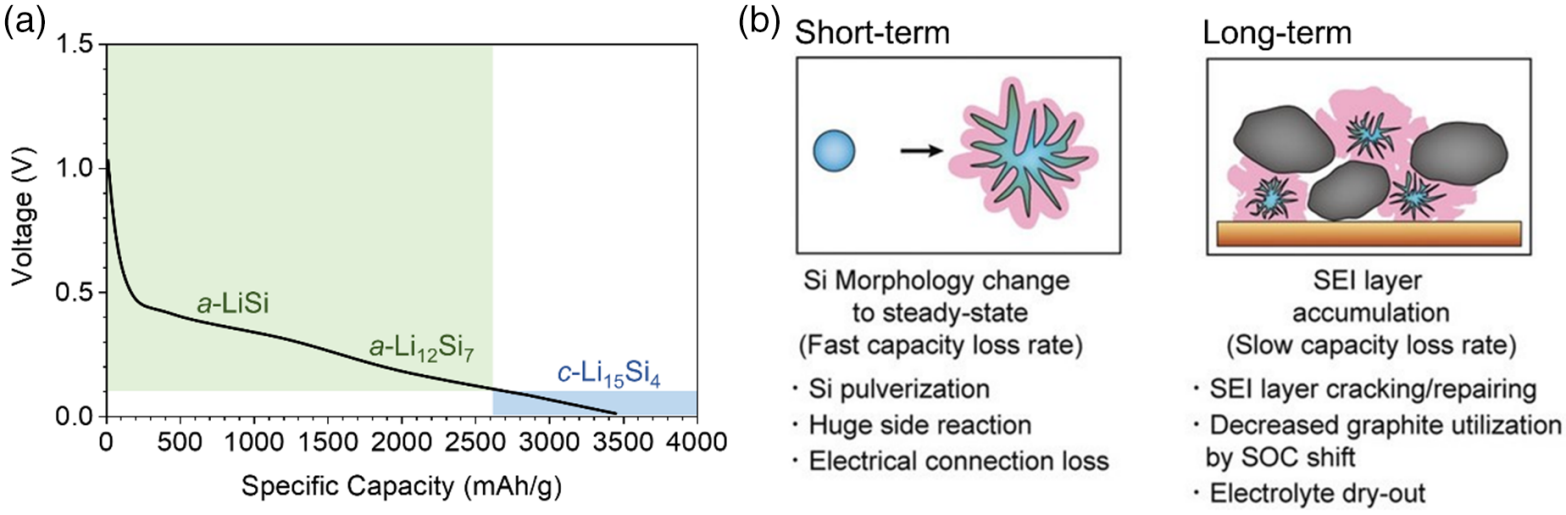
a) Lithiation of Si showing the different phases formed and their specific capacities. b) Failure mechanism of Si–graphite. b) Reproduced with permission.^[^ ^53^ ^]^ Copyright 2020, Wiley‐VCH.

Silicon forms a multitude of Li_*x*_Si compounds upon lithiation including LiSi, Li_12_Si_7_, Li_15_Si_4_, and Li_21_Si_5_, and the nature of the intermediate has a great impact on Li‐ion diffusion in Si. At potentials >0.07 V (vs. Li/Li^+^), amorphous intermediates (LiSi, Li_12_Si_7_) are formed, whereas crystalline Li_15_Si_4_ is formed below 0.07 V (**Figure** **3**a).^[^ ^50^ ^]^ Previous results have shown that each of these Li_*x*_Si compounds has a high Li‐ion diffusion coefficient in the range 10^−7^–10^−9^ cm^2^ s^−1^.^[^ ^51^ ^]^ However, the phase transition from Si to crystalline Li_*x*_Si is accompanied by a high activation energy of ≈3.13 eV,^[^ ^50^, ^52^ ^]^ which makes the formation of crystalline Li_15_Si_4_ and Li_21_Si_5_ difficult. After being fully discharged, the final product is Li_15_Si_4_ instead of Li_21_Si_5_, indicating that phase transition to the crystalline phase is far from easy. This could be the reason for the low experimentally measured value for Li‐ion diffusion coefficient of 10^−14^–10^−16^ cm^2^ s^−1^ for the Si‐based anode.^[^ ^12^ ^]^ These results indicate that the sluggish phase transition from amorphous to crystalline phase is unfavorable for Li‐ion diffusion and may be the main hindrance to achieving fast lithiation of silicon.

In addition to the limitation in lithiation kinetics, the accompanied volume expansion also poses a problem when using Si anodes. Silicon suffers a ≈400% volume increase upon lithiation, which increases the contact area between the pulverized Si anode and the electrolyte and results in further electrolyte decomposition, leading to an additional SEI generated on the surface of the pulverized silicon. A thick SEI can disrupt electrical contact between silicon and graphite and also gives rise to a large impedance to Li‐ion conduction (Figure 3b).^[^ ^53^ ^]^ Although the fracturing of the Si anode does not pose a problem at low current density, the high Li‐ion conduction impedance and the low electrical conductivity of Si inhibit Li ions from crossing the SEI and alloying with Si at high current density. Thus, under fast charging conditions, the Si anode can easily lose electrochemical activity. To accommodate the volume expansion of Si, graphitic materials with different microstructures have been used as matrices to form composites.^[^ ^54^ ^]^ Nevertheless, the capacity retention of Si/graphite anode at high rates is still far lower than what is required for XFC up to now.

In addition, SiO_*x*_ and graphite mixture were also demonstrated as an anode material for LIBs. The lithiation products of SiO_*x*_ have Li_4_SiO_4_ and Li_2_O in addition to Li_*x*_Si. The Li_4_SiO_4_ acts as an effective buffer for the volume changes, which reduces the volume change from 300% (Si) to 160% (SiO_*x*_).^[^ ^55^ ^]^ Li_4_SiO_4_ and Li_2_O also show high a Li‐ion diffusion coefficient of 10^−9^ and 10^−7^ cm^2^ s^−1^.^[^ ^56^ ^]^ The merit ion conductivities of Li_4_SiO_4_ and Li_2_O enhance the Li‐ion diffusion into the nanosilicon domains by creating additional phase boundaries within the mixed structure and further improving the rate performance of SiO_*x*_. However, the formation of irreversible Li_4_SiO_4_ and Li_2_O means the low initial Columbic efficiency (ICE), which is only 50–60%. To improve the ICE, the prelithiation technique can enhance the ICE as high as 90%.^[^ ^57^ ^]^

The average lithiation potential of Si is 0.2 V versus Li/Li^+^, which is very close to that of graphite. The sluggish electrochemical kinetics of Si also leads to a large overpotential under fast charging, which increases the risk of lithium plating. Most recent results have indicated that lithium plating occurs on the silicon/graphite anode at a low charge rate of 0.5C even though the n/p capacity ratio reaches 1.2.^[^ ^58^ ^]^ This result indicates that even after replacing the graphite anode with a Si/graphite anode, it is still difficult to avoid lithium plating. The recent research designed a robust Si/C microsphere with a compact nano‐/microstructure to achieve low volume expansion during cycling, which exhibited 85% capacity retention over 300 cycles at a high areal capacity of 4 mAh cm^−2^.^[^ ^59^ ^]^ In addition, reduced particle size can reduce volume expansion and shorten the Li^+^ diffusion path. The subnano‐sized Si/graphite demonstrated an outstanding rate capability of 79.6% capacity retention at 5C.^[^ ^8^ ^]^ To improve the electrochemical performance of SiO_*x*_, a recent report synthesized an in situ graphene‐coated disproportionated SiO (D‐SiO@G) anode, which presented a high rate capability of 774 mAh g^−1^ at 5 A g^−1^.^[^ ^60^ ^]^ Other carbon species also worked. By in situ growing of carbon nanotubes (CNTs) and graphitic carbon on the surface of SiO_*x*_, the as‐prepared SiO_*x*_@CNTs/C delivered a remarkable capacity of 327 mAh g^−1^ after 1000 cycles at 5 A g^−1^.^[^ ^61^ ^]^

Overall, Si/graphite is a widely studied anode material due to its several advantages. Nevertheless, the sluggish phase transition between amorphous and crystalline Li_*x*_Si compounds leads to slow Li‐ion diffusion and the accompanied huge volume expansion causes capacity loss under cycling. Thus, it is important to improve silicon's lithiation kinetics and structure stability in Si/graphite composites. Moreover, at the microlevel, the mechanism of lithium plating on the Si graphite surface needs to be clarified, which is key to achieving XFC safety.

## 4 Phosphorus‐Based Anodes

Phosphorus has three main allotropes, namely, white P, red P (RP), and black P (BP). Phosphorous alloys with three Li atoms to form Li_3_P deliver a theoretical capacity of 2596 mAh g^−1^. Following the first report by Park et al., RP and BP have been widely investigated as anode materials for LIBs.^[^ ^62^ ^]^ The average lithiation potential of phosphorus is 0.75 V Li/Li^+^.^[^ ^63^ ^]^ Though this high lithiation potential compromises the output voltage and thus the energy density of the battery, lithium plating can be inhibited, especially under fast charging conditions. As phosphorus is an alloy‐type anode material similar to silicon, we consider the fast charging performance of the phosphorus anode with respect to Li‐ion diffusion capability, electrical conductivity, and structural stability.

**Figure 4:**
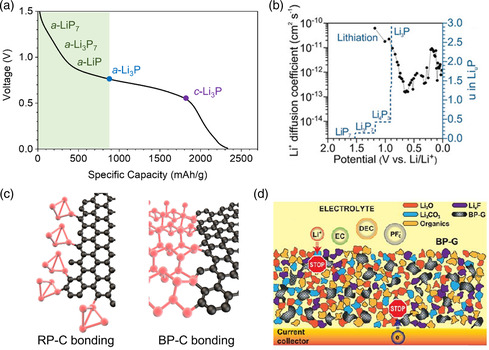
a) Phase evolution of P during lithiation. b) Li‐ion diffusion coefficient at different lithiation potentials, and the average potential between pairs of proximate stable lithium phosphides. c) Covalent P—C bonds between BP/RP and the carbon matrix. d) Polymer coating for the optimized SEI structure. b) Reproduced with permission.^[^ ^68^ ^]^ Copyright 2020, Wiley‐VCH. d) Reproduced with permission.^[^ ^76^ ^]^ Copyright 2020, American Association for the Advancement of Science.

During lithiation, phosphorus reacts with lithium to generate several different types of lithium phosphides (LiP_7_, Li_3_P_7_, LiP, and Li_3_P). These complex phase changes have a significant impact on Li‐ion diffusion in the P anode. More specifically, theoretical calculations indicate that the formation energy of Li_*x*_P is decreased with increasing *x* (*x* ≤ 3), and Li_3_P is formed around 0.82 V versus Li/Li^+^.^[^ ^64^ ^]^ Subsequent experiments also proved that amorphous Li_3_P is generated around 0.78 V and coexists with other amorphous Li–P compounds (LiP_7_, Li_3_P_7_, and LiP) before starting to transform to crystalline Li_3_P only at 0.54 V (**Figure** **4**a).^[^ ^65^, ^66^ ^]^ Moreover, due to the high ionic conductivity of Li_3_P,^[^ ^67^ ^]^ its very early generation can further facilitate the Li‐ion diffusion in the electrode. This is corroborated by the increase in Li‐ion diffusion coefficient from 10^−13^ to 10^−12^ cm^2^ s^−1^ after Li_3_P formation (potential below 0.7 V vs. Li/Li^+^, Figure 4b) and this value is unaffected by the crystallization process of Li_3_P (potential <0.5 V vs. Li/Li^+^).^[^ ^68^ ^]^ These results indicate that the phase transition behavior of phosphorus during lithiation is favorable to Li‐ion diffusion, which could, in principle, satisfy the requirements of fast lithium storage.

Similar to the Si anode, the P anode shows variable electrical conductivity (300 S m^−1^ for BP and 10^−12^ S m^−1^ for RP) and suffers ≈300% volume expansion during the alloying process. Fracturing of the active particle due to SEI generation results in phosphorus losing electrical contact with the conducting carbon. Indeed, engineering P into nanodimension and combining P with conductive carbon matrix are effective ways to conquer the volume expansion, enhance electronic conductivity, accelerate Li‐ion diffusion, and minimize the charge transfer resistance.^[^ ^69^ ^]^ Porous carbon and low‐dimensional carbon (such as graphene, carbon nanotube, mesoporous carbon, etc.) are used as P holders, and high‐energy ball milling or vaporization condensation methods were used to obtain uniformly mixed P/carbon composites at microscale.^[^ ^70^, ^71^, ^72^ ^]^ The carbon matrix constructs an electronic conductive network, which significantly enhances the electrical conductivity of P (especially RP), and nanosized P reduces the charge transfer resistance in the electrolyte/P interface and P/carbon matrix. Moreover, these carbonaceous materials with high specific surface areas can offer extra space to accommodate the volume expansion of P during lithiation.

Interestingly, phosphorus can form phosphorus—carbon (P—C) bonds with the carbon matrix when subjected to high‐energy input during mixing (Figure 4c).^[^ ^19^, ^73^ ^]^ A covalently bonded interface has been more widely observed in phosphorus/carbon composites than in other carbon‐based composites, for example, the Si/carbon matrix.^[^ ^53^, ^74^ ^]^ This may due to the bond energy of the P—C bond (264 kJ mol^−1^), which is much lower than that of the Si—C bond (360 kJ mol^−1^).^[^ ^75^ ^]^ The formation of covalent P—C bonds maintains a robust phosphorus‐carbon interface, which is critical to withstanding volume expansion‐induced stress, which in turn prolongs cycling life. Our recent studies have shown that the presence of the P—C bond enhances the Li‐ion diffusion coefficient by an order of magnitude in the composite as compared with pristine P.^[^ ^76^ ^]^ Furthermore, the P—C‐bonded interface enhances the electrical conductivity and improves the structure stability of the composite.^[^ ^18^, ^77^, ^78^, ^79^ ^]^

In view of the above‐mentioned advantages including favorable Li‐ion diffusion properties, improved electrical conductivity, and structural stability gained from P—C bonding, P–carbon composite materials appear to be ideally suited for XFC. However, due to the high average working potential of phosphorus, the P content in the P–carbon composite should be high enough (>50 wt%) to obtain a gravimetric energy density comparable with that of a full cell using a Si/graphite anode. However, increasing the P content in P/C‐based composites is complicated by the following issues. The bonding efficiency of P and C is quite low when using mechanical ball milling or vaporization condensation method, which might reduce the number of P—C bonds in the P/C composite and have a negative impact on its structural stability. Therefore, new methods to enable efficient bonding of P and C need to be developed. Moreover, most recent research results have shown that the lithium polyphosphide intermediates may be soluble in electrolyte,^[^ ^80^ ^]^ which may accelerate capacity fading upon cycling. Finally, the high P content also increases SEI generation, which affects both Li‐ion diffusion and electron conductivity. Thus, to maintain ionic/electron transfer and structural stability, it is important to appropriately modify the surface composition of the P–C hybrid particles to prevent increase in SEI caused by particle pulverization.

It is found that interface modulation does help in resolving these issues. A recent study reported a P‐based composite with an improved P–C interphase fabricated by combining 0D Ketjenblack and 1D carbon nanotubes. The composite exhibited a high ICE of 91%, high reversible capacity of 1750 mAh g^−1^, and high capacity retention of 71.2% at the rate of 2.4C. This improved performance was attributed to the ability of the composite structure to effectively accommodate volume changes, suppress irreversible electrolyte decomposition, and enable highly reversible phase changes.^[^ ^81^ ^]^ Another study noted that the presence of a polymer coating on the P–carbon composite particle (P content >60 wt%) can prevent active particle cracking and the polymer also formed a part of the SEI to boost ionic/electron transport (Figure 4d).^[^ ^76^, ^78^ ^]^ The polymer‐coated phosphorus‐based composite showed a capacity retention of 75% at ≈3C at an anode areal capacity of 2.2 mAh cm^−2^. Moreover, combining a 3D framework conductive CNT matrix with higher Li adsorption energy N doping, the RP/CNG composite exhibits a great rate capability of 1340.5 mAh g^−1^ at 3.9 A g^−1^.^[^ ^82^ ^]^ Other research reported a black phosphorus/Ketjenblack‐MWCNTs(BPC), which showed a high ICE of ≈91% and an excellent capability of ≈1600 mAh g^−1^ at 6.24 A g^−1^, and good cycle stability of ≈88% capacity retention after 100 cycles when combined with LiNi_0.6_Co_0.2_Mn_0.2_O_2_ in full‐cell testing.^[^ ^81^ ^]^ The latest study also showed that by implanting Bi with a low Li^+^ diffusion barrier and high electrochemical activity into a P/graphite (P/C) composite, the Bi–P/C anode provided a high fast charging capacity of 1788.2 mAh g^−1^ at 13 A g^−1^.^[^ ^83^ ^]^

In addition, introducing additives to the electrolyte helps to form a thin stable SEI coating on the surface of the active particles. For instance, during lithiation, the fluoroethylene carbonate (FEC) additive decomposes before ethylene carbonate and the decomposition products of FEC (a part of the SEI) are uniformly coated on the surface of P–C particles prior to the lithiation reaction.^[^ ^84^, ^85^ ^]^ The as‐formed SEI favors Li‐ion diffusion and further enhances capacity retention at high rates. These results demonstrate that interface optimization can significantly improve the rate performance and cycling stability of P–carbon composites with high P content. Moreover, recent research found that the LiFSI–TEP:FEC electrolyte system can improve the stability and rate performance of P anode. The electrolyte helps to form LiF‐rich and Li_*x*_PF_*y*_‐less SEI, which conquer the volume change and boost Li‐ion diffusion through SEI. The RP‐based anode exhibits high capacity retention of ≈600 mAh g^−1^ at 7 A g^−1^ and ≈300 mAh g^−1^ after 300 cycles at 3 A g^−1^.^[^ ^85^ ^]^

In conclusion, P–carbon‐based materials show favorable lithium‐ion diffusion ability and electronic conductivity. However, the high average lithiation potential of the P–carbon anode reduces the energy density of the full cell. To meet the energy density requirements of current LIBs, the phosphorus content in the P–carbon composite anode must be increased to >50 wt%. This leads to the accumulation of SEI on the electrode surface, which affects Li‐ion diffusion and destabilizes the electrode. Although P—C bonding can improve the stability of the electrode to a certain extent, the electrochemical performance is still not sufficiently high for fast charging. A modulation of the interface, reportedly, enhances ion transport at the interface and increases the stability of the electrode, thereby achieving satisfactory fast charging performance.

## 5 Perspectives

In this article, we discussed the factors that influence the fast charging performance of LIBs when using the current mainstream anode materials. Among the various anode materials, graphite has the lowest lithiation potential and best structural stability. However, when using graphite, the SEI formed can block single‐ion diffusion channels and hinder the transport of lithium ions. This increases the overpotential and results in lithium plating during fast charging. Si/graphite anodes have high capacity, but the difficult phase transition from amorphous Li_*x*_Si to crystalline Li_15_Si_4_ limits lithium‐ion diffusion. In addition, the accompanied volume expansion causes silicon to lose electrical contact with the conducting carbon resulting in loss of capacity. Silicon anodes are still a long way from achieving fast lithium storage performance.

Phosphorus has a high theoretical capacity, favorable phase transition, and easily forms stable chemical bonds with the carbon matrix and has therefore great potential for fast charging LIB anode application. The average lithiation potential of P is 0.7 V, which helps to avoid lithium plating under XFC conditions, but the low potential also reduces the energy density of the full cell. Consequently, the P content needs to be increased to over 50 wt% to achieve energy densities comparable with that of current LIBs. Increased P content results in SEI accumulation, which leads to a sluggish Li‐ion diffusion across the SEI.

In this scenario, we believe that interface optimization of the P–carbon composite material can enhance fast charging performance. Thus, future research on P‐based anodes for fast charging LIBs should mainly focus on the following aspects. 1) Improving the ICE of P–carbon anode; 2) clarifying the mechanism of how the formation of a chemical bond (P—C or P—O—C bond) enhances the structural stability and charge transfer during lithiation/delithiation; 3) designing new preparation methods to improve P—C bonding in composites with a high P content; 4) estimating the highest rate performance that can be obtained without lithium plating for P–C anode electrodes in a full cell; 5) finding suitable electrolytes that can form a stable SEI at the interface to boost charge transfer and maintain the stability of the P–C anode during fast charging; and 6) finally, the development of large‐scale preparation methods for P–carbon composite anodes that would make its industrial‐scale application viable.

## Conflict of Interest

The authors declare no conflict of interest.
